# The microphysics of surrogates of exhaled aerosols from the upper respiratory tract

**DOI:** 10.1080/02786826.2023.2299214

**Published:** 2024-01-17

**Authors:** Jianghan Tian, Robert W. Alexander, Daniel A. Hardy, Thomas G. Hilditch, Henry P. Oswin, Allen E. Haddrell, Jonathan P. Reid

**Affiliations:** a School of Chemistry, University of Bristol, Bristol, UK; b School of Cellular and Molecular Medicine, University of Bristol, Bristol, UK; c School of Earth and Atmospheric Sciences, Queensland University of Technology, Brisbane, Queensland, Australia

## Abstract

Airborne transmission plays a significant role in the transmission of respiratory diseases such as COVID-19, for which the respiratory aerosol droplets are responsible for the transportation of potentially infectious pathogens. However, the aerosol physicochemical dynamics during the exhalation process are not well understood. The representativeness of respiratory droplet surrogates of exhaled aerosol and suspension media for aerosols currently used for laboratory studies remains debated. Here, we compare the evaporation kinetics and equilibrium thermodynamics of surrogate respiratory aerosol droplets including sodium chloride, artificial saliva (AS) and Dulbecco’s modified Eagle’s medium (DMEM) by using the Comparative Kinetics Electrodynamic Balance. The potential influences of droplet composition on aerosol hygroscopic response and phase behavior, and the influence of mucin are reported. The equilibrium hygroscopicity measurement was used to verify and benchmark the prediction of evaporation kinetics of complex solutions using the Single Aerosol Particle Drying Kinetics and Trajectory model. We show that the compositionally complex culture media which differs from sodium chloride and artificial saliva (mucin-free solutions). The DMEM evaporation dynamics contained three distinctive phases when drying at a range of humidities, including a semi-dissolved phase when evaporating at the environmental humidity range. The effect of mucin on droplet evaporation and phase behavior at low RH were compared between AS and DMEM solutions. In both cases, mucin delayed the crystallization time of the droplets, but it promoted phase change (from homogenous to semi-dissolved/spherical with inclusions) to occur at higher water activities.

## 1 Introduction

Respiratory aerosol droplets are responsible for the transmission of respiratory diseases such as COVID-19 (Jayaweera et al. 2020; Pöhlker et al. 2021; Wang et al. 2021; Tang, Tellier, and Li 2022). An understanding of the mechanisms of respiratory aerosol transformation during exhalation, improved quantification of the amount of aerosol generated and the particle size distributions, and identification of the particle sizes that carry the pathogen load are all important for improving models of transmission (Fernstrom and Goldblatt 2013; Vuorinen et al. 2020; Bagheri et al. 2023; Morawska et al. 2009). Once exhaled, the microphysical processes that control particle size, water content and composition may all play a role in the transmission distance and pathogen survival in inhalable particles (Walker et al. 2021; Oswin et al. 2022). Indeed, the interaction between microorganisms within aerosol droplets and the ambient environment (e.g., relative humidity and temperature) has been investigated by various experimental methods over many years. Various factors have been assessed to influence pathogen viability in the environment, including the composition of the droplet medium in which a pathogen is suspended, the formation of reactive oxygen species (Oswin et al. 2023), exposure to UV light, the ambient relative humidity (RH) and the temperature (Dabisch et al. 2021; Dabisch et al. 2022; Fernandez et al. 2019, 2020; Lin and Marr 2020; Schuit et al. 2020; Tang 2009). However, there is not a unified understanding of the mechanisms that control pathogen viability in the environment across pathogen type. Further studies are required to explore the physicochemical characteristics and dynamics of respiratory aerosols upon exhalation.

In aerosol laboratory studies, a medium within which the pathogen is suspended must be selected. Artificial saliva (AS), Dulbecco’s modified Eagle’s medium (DMEM) and minimal essential medium (MEM) with clinically relevant mucin concentrations have all been used as suspension media in studies of viral survival in aerosols (Alexander et al. 2022; Oswin et al. 2022, 2021). However, a detailed understanding of the microphysical processes that transform the aerosol on generation and suspension is required for interpretation of bioaerosol viability and infectivity. Careful consideration of the composition of relevant fluids is needed to inform matrix selection for future studies. For example, many physical characteristics of respiratory aerosol remain unknown, with limited studies of their hygroscopicity and phase morphology under ambient conditions (Groth et al. 2023; Vejerano and Marr 2018; Johnson et al. 2011; Groth et al. 2021). In addition, mucin which is a major component of respiratory secretions, has been recognized to support viral survival (Wardzala et al. 2022; Vejerano and Marr 2018); and therefore must be included within the suspension media of aerosol studies to accurately recreate the molecular complexity of respiratory fluid. As a result, the representativeness of respiratory droplet surrogates for exhaled aerosol currently used for laboratory studies remains debated, and an understanding of the potential influences of composition and phase change on the survival of virus in the aerosol phase is incomplete.

In this study, we firstly explore the evaporation kinetics and equilibrium thermodynamics of three surrogate solutions for respiratory fluid from the upper respiratory tract, of different chemical compositions and complexities: sodium chloride (NaCl), artificial saliva (AS) and Dulbecco’s modified Eagle’s medium (DMEM). We will also compare these with our previous measurements of the properties of minimal essential media (MEM). Separately, we have used these media in studies of the airborne survival of SARS-CoV-2 and the Mouse Hepatitis Virus (Alexander et al. 2022; Oswin et al. 2021, 2022). The phase transformation and the effect of mucin concentration upon evaporation will also be reported. Although numerous studies have examined the equilibrium properties of surrogate respiratory fluid droplets with systematic variation in steady RH (Lin and Marr 2020; Luo et al. 2023; Huynh et al. 2022) or the drying kinetics of large microlitre droplets on surfaces (Lin, Schulte, and Marr 2020; Lin and Marr 2020), our aim is to explore the dynamical changes that occur during droplet drying, replicating the water transportation and pH changes that occur over short timescales when respiratory aerosol are exhaled from the warm humid lung into a dry atmosphere.

## 2 Materials and methods

### 2.1 Surrogate formulations

Sodium chloride, artificial saliva and Dulbecco’s modified Eagle’s medium solutions were prepared according to the following description. For convenience, the solutions will be referred to by the main components, simply as NaCl, AS and DMEM. In all experiments, HPLC-grade water was used. NaCl solution was made with a mass fraction of 0.1 (equivalent to 10% *w*/*w*, BioXtra ≥99.5%, Sigma-Aldrich). The artificial saliva formulation was reproduced following the procedure of Woo et al. (2010); the solution composition is summarized in Table S1. Additionally, based on the variation of dominant ions in saliva from unstimulated and stimulated saliva (including sodium, bicarbonate and chloride), an adjusted formulation was made by adding extra 0.4 g/L of NaCl and 4.5 g/L NaHCO_3_ based on the increased concentration of sodium, chloride, and bicarbonate reported by Thaysen, Thorn, and Schwartz (1954). This is referred to as artificial stimulated saliva (ASS) below.

The DMEM formulation used in this study is the same as Alexander et al. (2022). Briefly, Dulbecco’s modified Eagle’s medium (DMEM, high glucose; Sigma-Aldrich, St. Louis, MO, USA) is supplemented with 10% *v/v* fetal bovine serum (FBS, Sigma-Aldrich), and trace amount of penicillin, streptomycin and L-glutamine. The solution composition is summarized in Table S2. Type II porcine gastric mucin was used as a source of mucin and contains three different mucin proteins. Clinically relevant amounts of mucin are also added to DMEM to better simulate the nature of respiratory fluid and different respiratory tract states, specifically 0.1, 0.3, 0.5% *w*/*v*. % *w/v* represent the mass of mucin (in gram) dissolved in 100 mL of DMEM solution. The 0.1% *w/v* represents the mucin concentration in healthy individual, while 0.3 and 0.5% *w/v* were associated with mucin concentrations of respiratory fluid from smokers and individuals with asthma or COPD (Kesimer et al. 2017). For comparison with previous work, we included data from Eagle’s minimum essential medium plus GlutaMAX (MEM, Gibco, ThermoFisher, cat#41090036) supplemented with 2% *v/v* FBS and 0.1 mM non-essential amino acids (MEM 2% FBS), and the solution composition is summarized in Table S3.

### 2.2 Experimental method for studying the evaporation and phase state of surrogate respiratory fluid droplets

The evaporation kinetics of water from NaCl, AS and DMEM solution droplets were studied using the Comparative Kinetics-Electrodynamic Balance (CK-EDB) instrument. The instrument has been described in detail in previous work (Rovelli et al. 2016) and will only be briefly reviewed here. Charged aerosol droplets of known composition with highly reproducible initial radius of ~26 *μ*m are dispensed through a droplet-on-demand (DoD) dispenser (MicroFab®) into the EDB trapping chamber. From the respiratory aerosol size distributions, particles of 26 *μ*m radius are within the size range that is likely representative of particles expelled from vocalizing and coughing. Thus, simulated saliva is an appropriate matrix with a significant proportion of the expelled particles generated during a cough originating from the oral cavity (Harrison et al. 2023; Johnson et al. 2011). However, it should be noted that this particle size and the use of saliva as a matrix are not likely relevant to particles generated during normal quiet breathing, where particles are typically smaller than 1 *μ*m in diameter and are generated in the respiratory bronchioles from alveolar lining fluid (Johnson et al. 2011; Asadi et al. 2019; Larsson et al. 2017; Bredberg et al. 2012). A mixed flow of dry and wet nitrogen is passed over the trapped particle to change the surrounding relative humidity. The temperature of the gas flow is controlled by a circulating flow of ethylene glycol coolant through the electrode assembly through which the gas flow passes. The trapped droplet is illuminated by a 532nm laser and the resulting elastically scattered light pattern (phase function) is collected by a CCD camera that is centered at 45° to the forward direction of the laser (Haddrell et al. 2019). Measurements were typically made of the evaporation profiles of ~100 droplets drying at 40% RH. Through an analysis of light scattering and particle radius, the RH-dependent equilibrium phase and the time point at which the droplet scatters light consistent with a non-spherical or crystalline particle can be identified (Haddrell et al. 2019). Furthermore, the time point at which heterogeneity is first identified as forming in the evaporating droplet can be established from a change in scattered light intensity pattern consistent with spontaneous inclusion formation within the liquid host droplet.

Additionally, a falling droplet column (FDC) instrument was used to collect dry particles for further imaging analysis. A continuous stream of uniform droplets is injected into a vertical glass column with the RH and temperature maintained as the droplet train falls vertically down the column (Hardy et al. 2021). Dried particles of all compositions (NaCl, AS with and without mucin, DMEM with and without mucin) were collected on a glass slide at the bottom of the falling droplet column under the same RH and temperature conditions as the EDB measurement. The dried particles have also been analyzed by scanning electron microscopy (SEM). SEM produces images of different particle depth depending upon the method of imaging used: either collecting electrons with the backscattered electron detector (BED) or the secondary electron detector (SED). Images produced using the SED tend to reveal the surface structure of particles in most detail. Images produced using the BED typically reveal more subsurface structure but tend to have a higher level of noise, with a poorer resolution of particle edges against the deposition surface.

## 3 Results and discussion

We first present measurements of the evaporation and phase behavior of surrogate respiratory droplets of varying complexity. Secondly, we report studies of these same systems with the addition of mucin to explore the evaporation and phase behavior of aerosol in the presence of complex glycoproteins. DMEM and AS solutions do not fully represent real respiratory emission as they lack, for example, proteins and glycoproteins. However, a more representative surrogate for respiratory droplets can be achieved by adding mucins to DMEM and AS to simulate the complexity expected in real respiratory droplets.

### 3.1 Evaporation kinetics and equilibrium thermodynamics of mucin-free surrogate respiratory droplets

**Figure 1:**
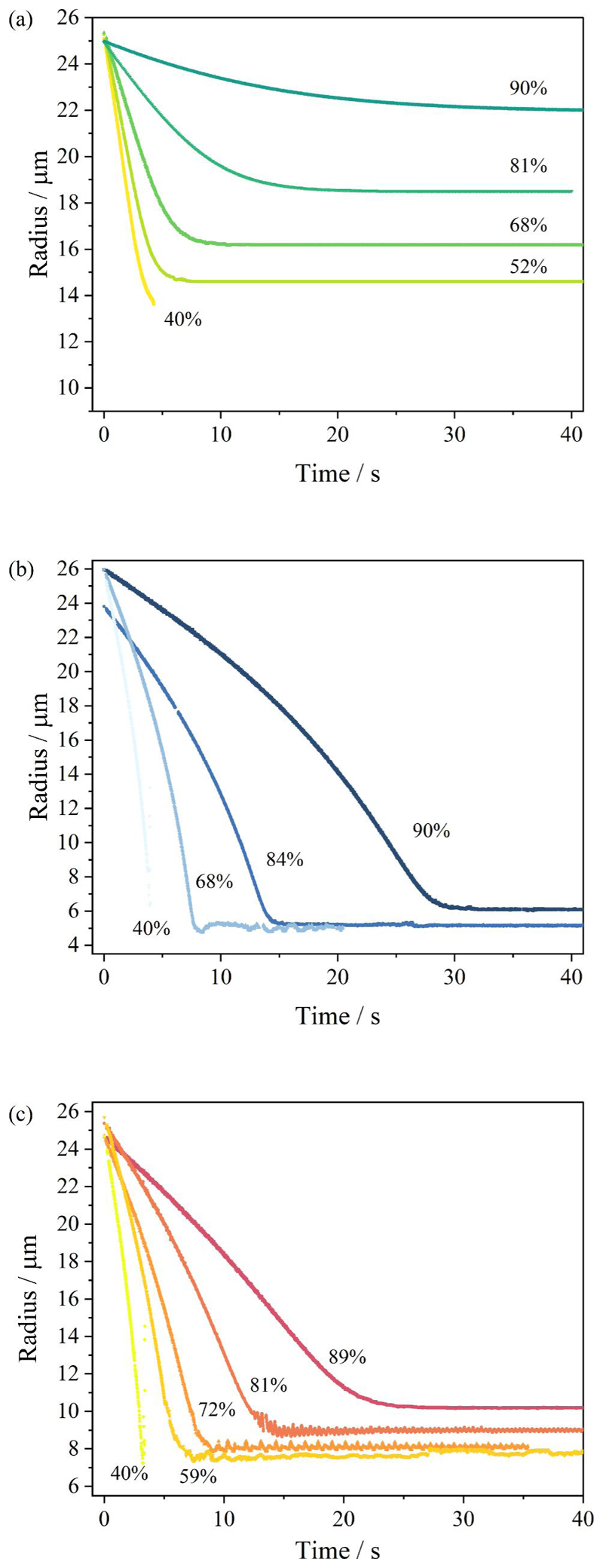
Evaporation profiles of (a) aqueous NaCl of 0.1 MFS concentration, (b) artificial saliva from (Woo et al. 2010) excluding mucin and DMEM, and (c) DMEM (with 10%FBS) droplets under different environmental conditions.

**Figure 2:**
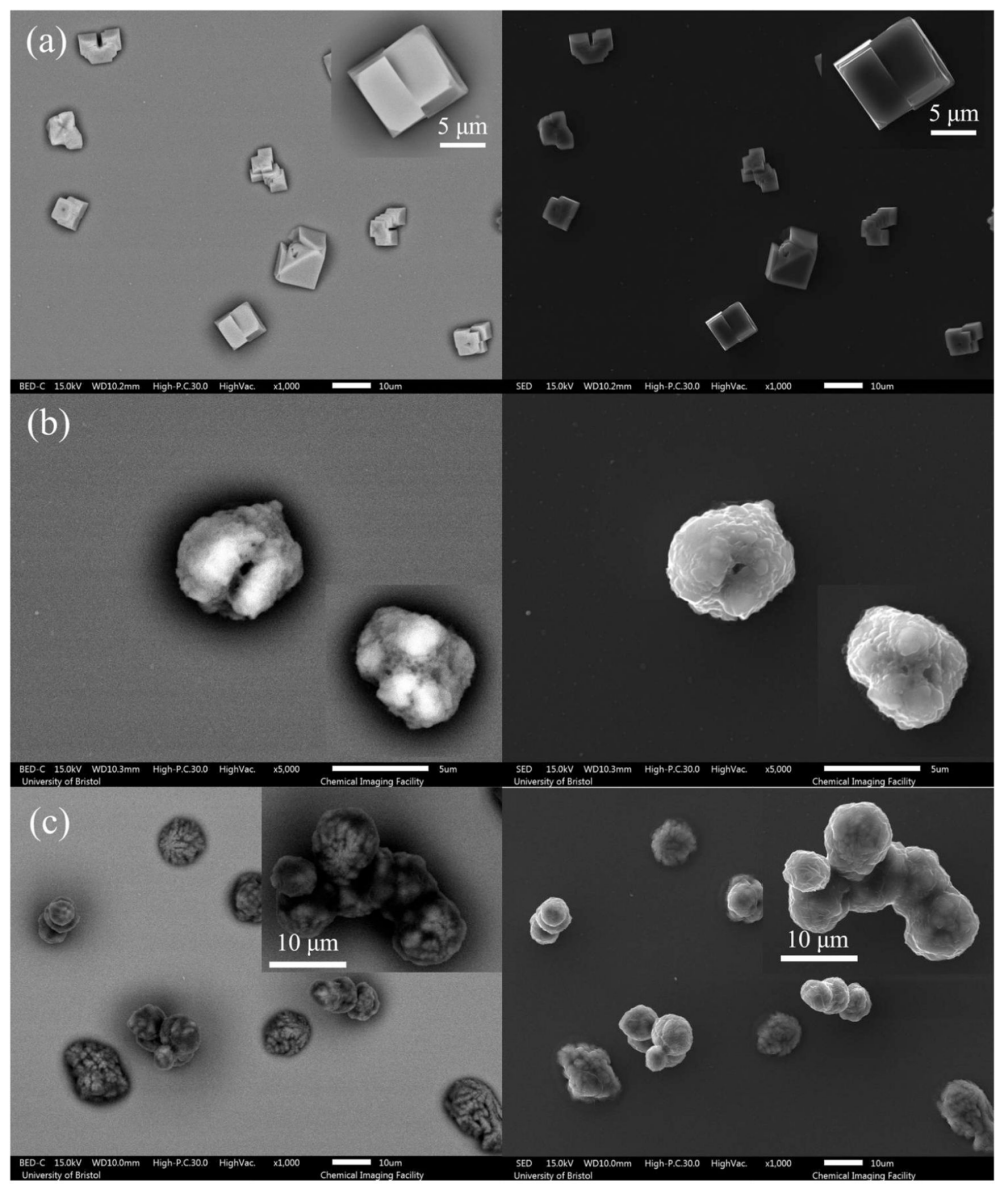
SEM images of (a) NaCl of 0.1 MFS concentration, (b) AS (non-mucin) from (Woo et al. 2010), and (c) DMEM (with 10% FBS, non-mucin) droplet drying at 40%RH. Note, each figure is composed of images from the backscattered electron detector (BED) (left) as well as the secondary electron detector (SED) (right), where the image produced by the BED reveals more subsurface structure. Typically, the number of backscattered electrons reaching the detector is proportional to the mean atomic number of the sample. In this case, we can clearly observe the brighter part from inorganic salt, and the darker parts from the organic fractions e.g., in (c) DMEM particles.

A qualitative comparison of the evaporation profiles (Figure 1) and morphology recorded by SEM (Figure 2) of the three studied surrogate solutions NaCl, AS and DMEM is provided in Figures 1 and 2. Droplet evaporation was studied under a range of relative humidities, from 40% to 90%. The evaporation profile of each solution at each RH are selected among 10 repeat measurements, the descriptive summary of the data and the ANOVA test of the final equilibrium size is attached in Table S4. Above efflorescence RH (an RH of ~43% for the three studied solutions), water within the droplet evaporates due to the higher initial water activity (*a*_*w*_ > 0.98) until it reaches equilibrium with the gas phase humidity, with the droplet size then remaining constant (Figure 1). The equilibrium droplet size is determined by the initial dry solute mass and the solute hygroscopicity (Fernandez et al. 2020). In this case, the hygroscopic response of three solutions cannot be inferred from a comparison of the equilibrium size of the droplet at varying RHs, since their starting mass fraction of solute are different (NaCl: 0.1; AS: 0.007; DMEM: 0.017). Below efflorescence RH, a phase change occurs forming a non-spherical inhomogeneous, amorphous or crystalline particle e.g., see particle dried at 40% RH in Figure 2. The light scattering from crystalline morphologies cannot be used to infer optical particle size due to the anisotropic scattering of light. Consequently, the size post-crystallization is not reported (Figure 1). It is also clear that different concentrations and compositions lead to different dried particle morphologies (Figure 2) with the much larger collection of inorganic salts presenting in AS and DMEM, causing significantly more nucleation sites within the droplets compared with NaCl.

**Figure 3:**
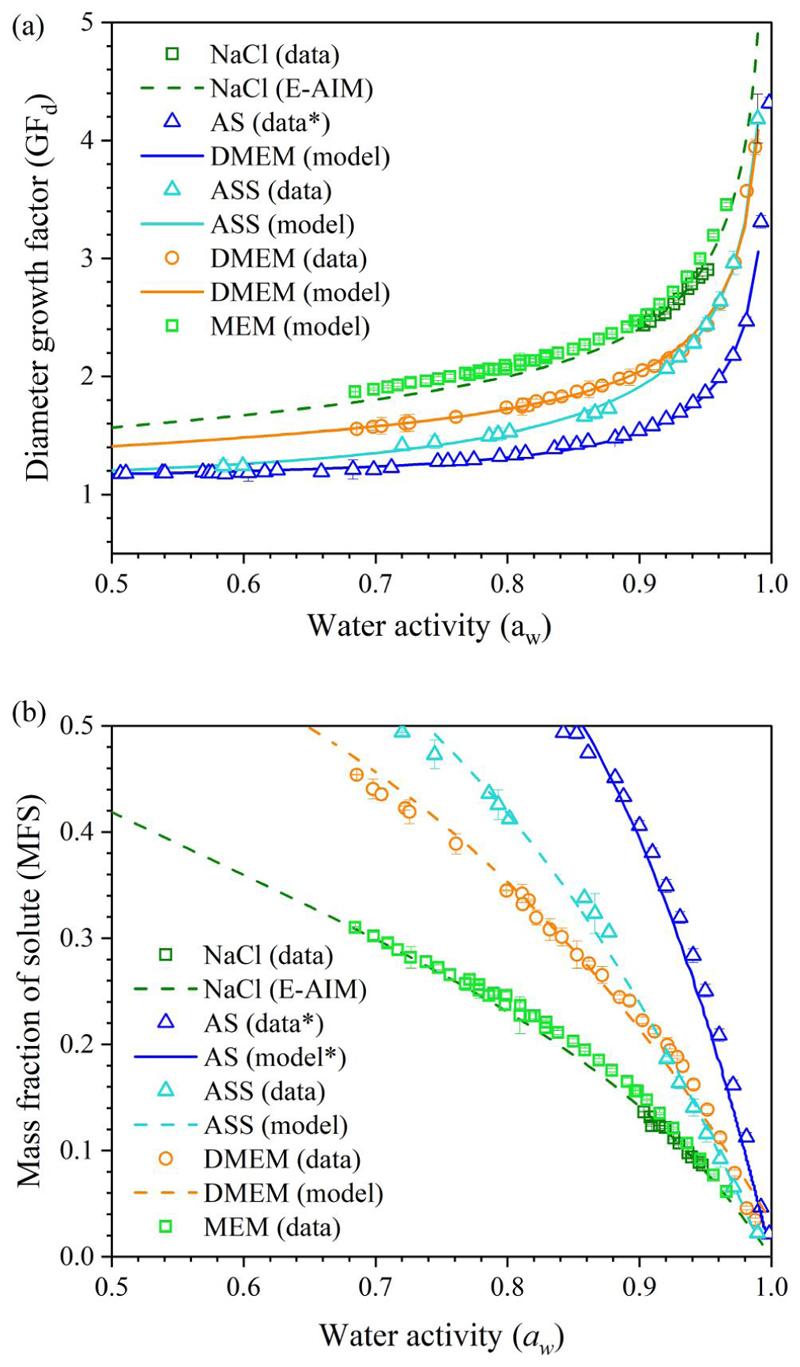
The hygroscopicity profile of surrogate solutions, including AS following Woo et al. (2010), DMEM with 10% FBS, MEM with 2% FBS and ASS formulation from adjusted AS recipe by increasing NaCl and NaHCO_3_ concentration. The fitting curve in (a) is included in Table S5 and the fitting curve for (b) is included in Table S6.

From kinetic measurements of the form reported in Figure 1, the equilibrium hygroscopicity response can be estimated for different surrogate solutions using our comparative kinetic approach (Haddrell et al. 2014; Marsh et al. 2019; Rovelli et al. 2016). The amount of residual water in an equilibrated droplet depends on its hygroscopic response, the variation in solution composition with water activity in the condensed phase. The expression of hygroscopicity of a given solution can be presented in two ways, either as growth factor (e.g., the ratio of wet and dry radii of the particle under humid and dry conditions) or as a mass fraction of solute (MFS) with changing water activity (equivalent to equilibrium RH). The diameter growth factors shown in Figure 3a can be converted to the MFS (Figure 3b), correcting the volume equivalent size from the growth factor to a relative mass ratio by using a parameterization of the solution density with square root of the MFS of the droplet. The density parameterisations of the solutions are summarized in Table S6.

The hygroscopic responses of aerosols formed from different media, inferred from CK-EDB measurements, are reported in Figure 3. In decreasing order of hygroscopicity (i.e., decreasing levels of absorbed moisture at a specific RH), the media follow the trend NaCl ~ minimal essential media (MEM) > DMEM > AS. The diameter growth factor of NaCl is greater than 2 when the RH is above 75% (Figure 1a). With the lowest growth factor, AS tends to equilibrate at a smaller size, which is only slightly larger than the dry size (Figure 1b). In the AS formulation, most solute mass comes from potassium chloride (KCl), sodium chloride and mucin. In the DMEM solution, NaCl, sodium bicarbonate (NaHCO_3_) and glucose are the major contributors to the solute mass; moreover, the presence of the organics reduced the overall hygroscopicity compared with NaCl solution. MEM is a cell culture medium that contains a lower organic fraction than DMEM and, in fact, shows a similar hygroscopicity to NaCl. In addition, artificial stimulated saliva (ASS) is included separately in Figure 3, showing a higher degree of hygroscopicity than AS due to the higher salts fraction. The kinetic evaporation profile of ASS is compared with AS in Figure S1.

The equilibrium solute concentration and ionic strength of surrogate droplets can be expected to impact on the viability of microorganisms included within the aerosol phase. Thus, knowledge of the hygroscopicity is vitally important to interpret and predict the viable lifetime of bacteria and viruses emitted in respiratory aerosol as a function of RH (Fernandez et al. 2019, 2020; Pöhlker et al. 2021).

**Figure 4:**
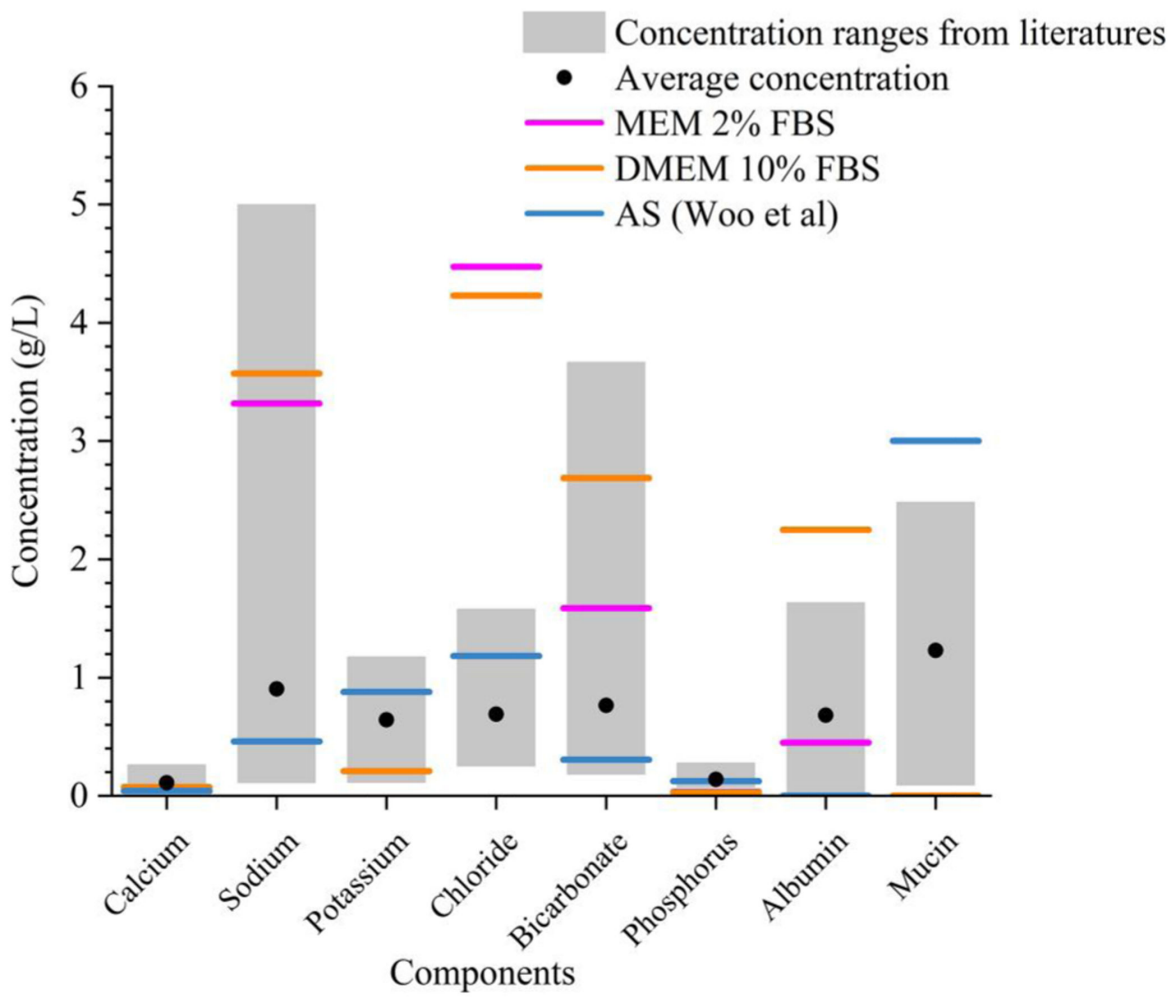
The concentration ranges of various components of human saliva and cell culture media.

Having underlined the importance of aerosol composition, recognizing the inter-person and diurnal variability of the major ion concentrations in human saliva is also important. In the absence of direct measurements of the composition of exhaled respiratory aerosol, the aerosol is often assumed to have the same composition (or at least contain the same components) as saliva. Figure 4 summarizes the concentration range of major ions in human saliva and compares these to their concentrations in various surrogate solutions. The grey bar indicates the variation of the ion concentrations across different studies (Thaysen, Thorn, and Schwartz 1954; Kallapur et al. 2013; Chauncey et al. 1962; Chauncey 1955). It is challenging to reflect the true nature of the variability of chemical composition in human saliva with surrogate formulations, but it is possible to provide a snap shot of the saliva states by presenting the stimulated saliva level and unstimulated saliva level. The artificial saliva recipe used in the EDB measurements is more compositionally relevant to unstimulated saliva, with much lower NaHCO_3_ and NaCl concentration than expected in stimulated saliva (Woo et al. 2010). For the ASS, normally it is observed with a higher flow rate and higher concentration of ions compared to unstimulated saliva (Bardow et al. 2000) through stimuli (e.g., eating), so the AS recipe (Woo et al. 2010) was adjusted by adding 4.5 g/L NaHCO_3_ (4.92 g/L in total) and 0.4 g/L NaCl (1.28 g/L in total) based on (Thaysen, Thorn, and Schwartz 1954). The recognition of the highly variable concentration and composition in the nature of the saliva is a significant step in understanding the connections between airborne viral viability and aerosol composition. The investigation of variation in saliva compositions is needed to account for the person-to-person variability. Future work could create bespoke formulations that mimic a range of saliva compositions. In addition, it is also important to note that the lung fluid and sputum are also examples of respiratory fluids that vary between individuals (Bredberg et al. 2012; Larsson et al. 2017). It is possible to investigate the role of composition for these fluids by using a similar approach.

**Figure 5:**
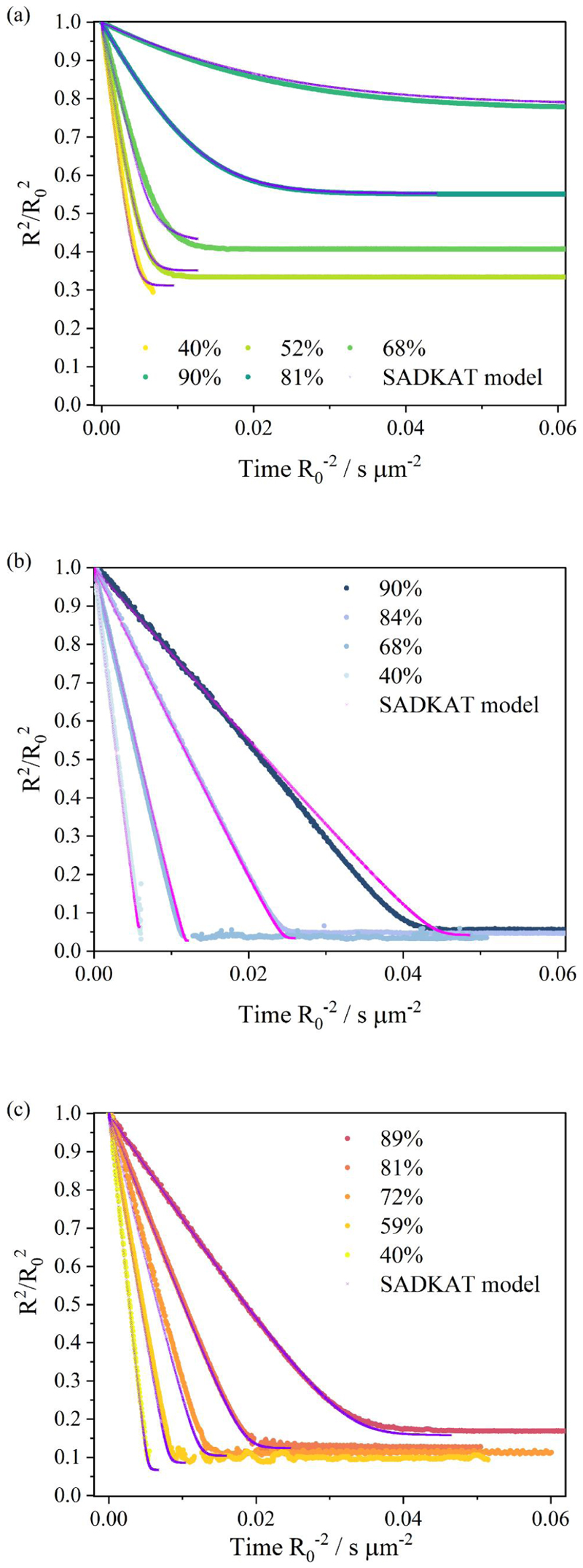
Comparison of evaporation profiles of SADKAT model and EDB measurement in (a) aqueous NaCl solution with 0.1 MFS concentration; (b) AS without mucin from (Woo et al. 2010) and (c) DMEM (with 10%FBS) solution droplets.

By retrieving the hygroscopicity profiles of a surrogate solutions, we are able to predict the evaporation kinetics of any surrogate solution under any set of environmental conditions and for particles of any size. But here, we focus on the single particle measurements of similar initial droplet size (~26 *μ*m) but of different chemical composition, and the particle size of interests are within the Laryngeal/Oral mode (Johnson et al. 2011). As verification, we compare our EDB measurements with simulations from the “Single Aerosol Drying Kinetics and Trajectories (SADKAT)” model using the equilibrium hygroscopic response as input to the model (Hardy et al. 2023) (Figure 5). We label the simulations generated using the model as “SADKAT” throughout for simplicity. SADKAT can be used to calculate evaporation and condensation rates of volatile components (e.g., water as a solvent) over the full range of environmental conditions; previously, it has been used for predicting the drying kinetics of aqueous solutions of salts and organics (Hardy et al. 2023). The comparison of measurements and simulations confirms that the SADKAT model can predict evaporation kinetics of simple solutions and complex solutions with high accuracy. SADKAT incorporates explicitly the coupling between heat and mass transfer, including the effects of evaporative cooling. It does not include explicitly the internal concentration gradients which can be established within droplets evaporating at high Peclet number. Indeed, a minor divergence of predictions from the measured trends is observed as the droplets approach equilibrium, possibly as a consequence of surface enrichment (Gregson et al. 2019).

**Figure 6:**
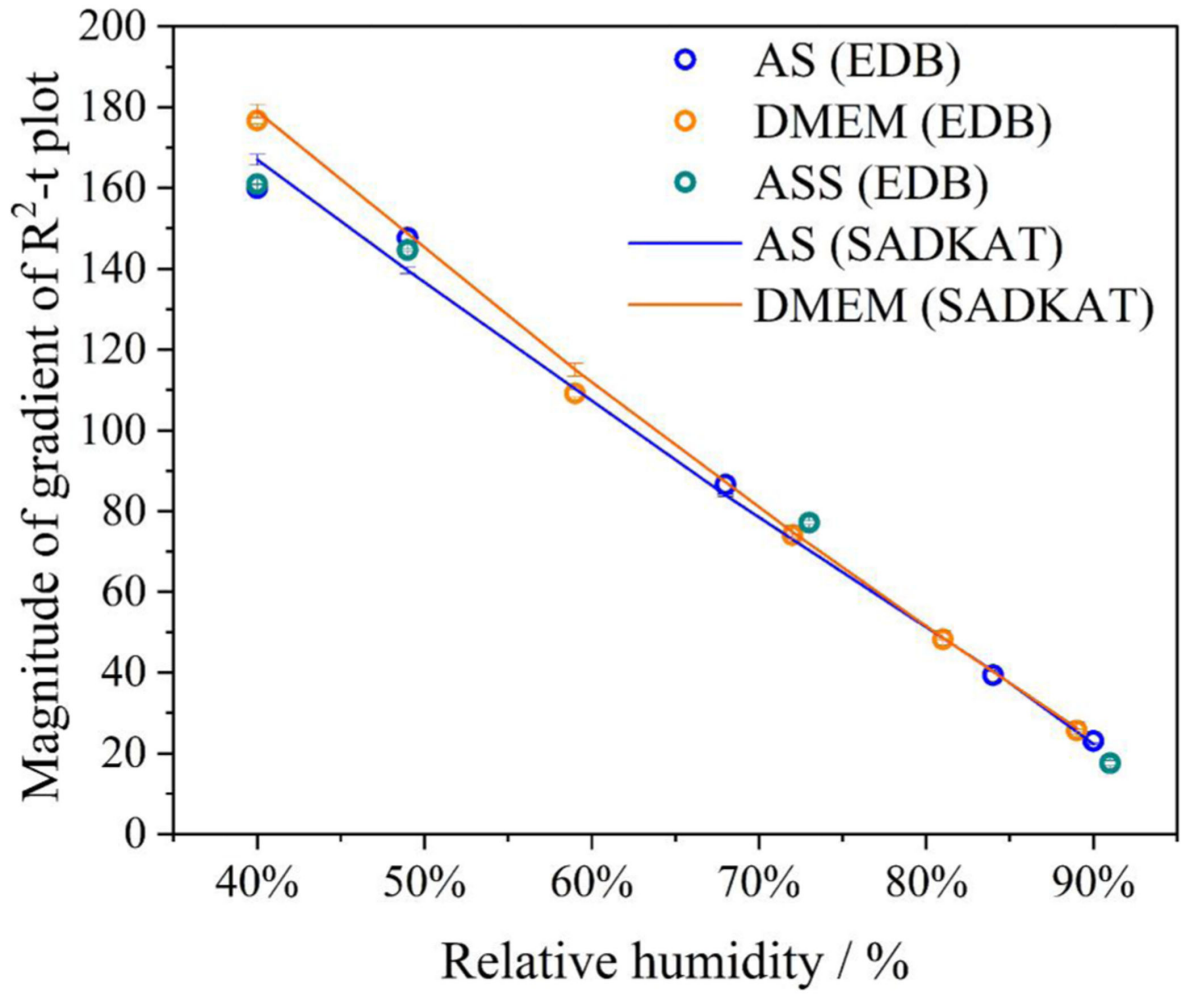
The RH-dependence of evaporation rate for different solution droplets compared to SADKAT model.

The RH dependent evaporation rates for NaCl, AS and DMEM were compared with one another and with the SADKAT model (Figure 6). Overall, the evaporation rate of AS and DMEM in different RHs are comparable and, notably, the two solutions started at similar water activity from 0.983 (DMEM) to 0.993 (AS). SADKAT treats mixed solutions as a single involatile component. The measurements and model predictions are in good agreement at the higher RH values shown, but diverge marginally under drier conditions as the relative hygroscopicities of the solutions diverge more appreciably. Again, fast evaporation in RHs below 40% leads to surface enrichment (e.g., core-shell structure) for certain chemical compositions (e.g., organics) in the droplet, which makes predictions of the evaporation using SADKAT less robust.

### 3.2 Phase transformation during evaporation of surrogate respiratory droplets

The humidity-dependent phase transformation of respiratory droplets have an impact on viral and bacterial survival in the aerosol phase (Fernandez et al. 2019, 2020; Haddrell et al. 2023; Huynh et al. 2022; Oswin et al. 2022); it is crucial to understand how RH impacts the phase behavior of aerosol particles for culture media and surrogate respiratory droplets. The complex phase behavior of an evaporating droplet contributes to increased variability in microbial viability. The virus or bacteria distribution within the evaporating droplet can impact viral/bacterial longevity. For example, the solid carbohydrates, proteins, or multiple salt crystals, could offer protective effects. Alternatively, in the liquid semi dissolved phase, an increase in pH could decrease viability. A recent study recognized the aerosol pH effect on viral survival (Haddrell et al. 2023). This also raises the question about the pH distribution within the droplet in combination with the phase change and its corresponding effect on viral/bacterial survival.

The identification of non-spherical inhomogeneous/crystalline, semi-dissolved (spherical with inclusions) and homogenous spherical (well-mixed) particle phases from singly levitated aerosol particle measurements is based on analysis of the angularly resolved phase function and the total elastic scattered light intensity (Haddrell et al. 2019). A semi-dissolved (or a spherical droplet with inclusions) state is identified as a droplet that remains spherical in shape but contains multiple crystals within the droplet. The droplet size can still be estimated because the angles of the light scattering peaks remain unaltered by the presence of the inclusions in the phase function, but irregularities in intensity across the angular range occur (Haddrell et al. 2019). It is important to note that the laser scattering method and three proposed phases do not necessarily capture all the nuanced details of structures that form on evaporation, but that the dominant structure classifications can be identified. For the core-shell phase identification, the sensitivity of the algorithm was found to be sufficiently sensitive to detect a monolayer of thickness >0.0025 *μ*m; for inclusions, the algorithm will correctly identify the particle as a sphere with inclusions when the total volume fraction of inclusions in the droplet is greater than 0.04% (for 450 nm spheres) (Haddrell et al. 2019). A droplet is assigned as homogeneous when below these limits.

**Figure 7:**
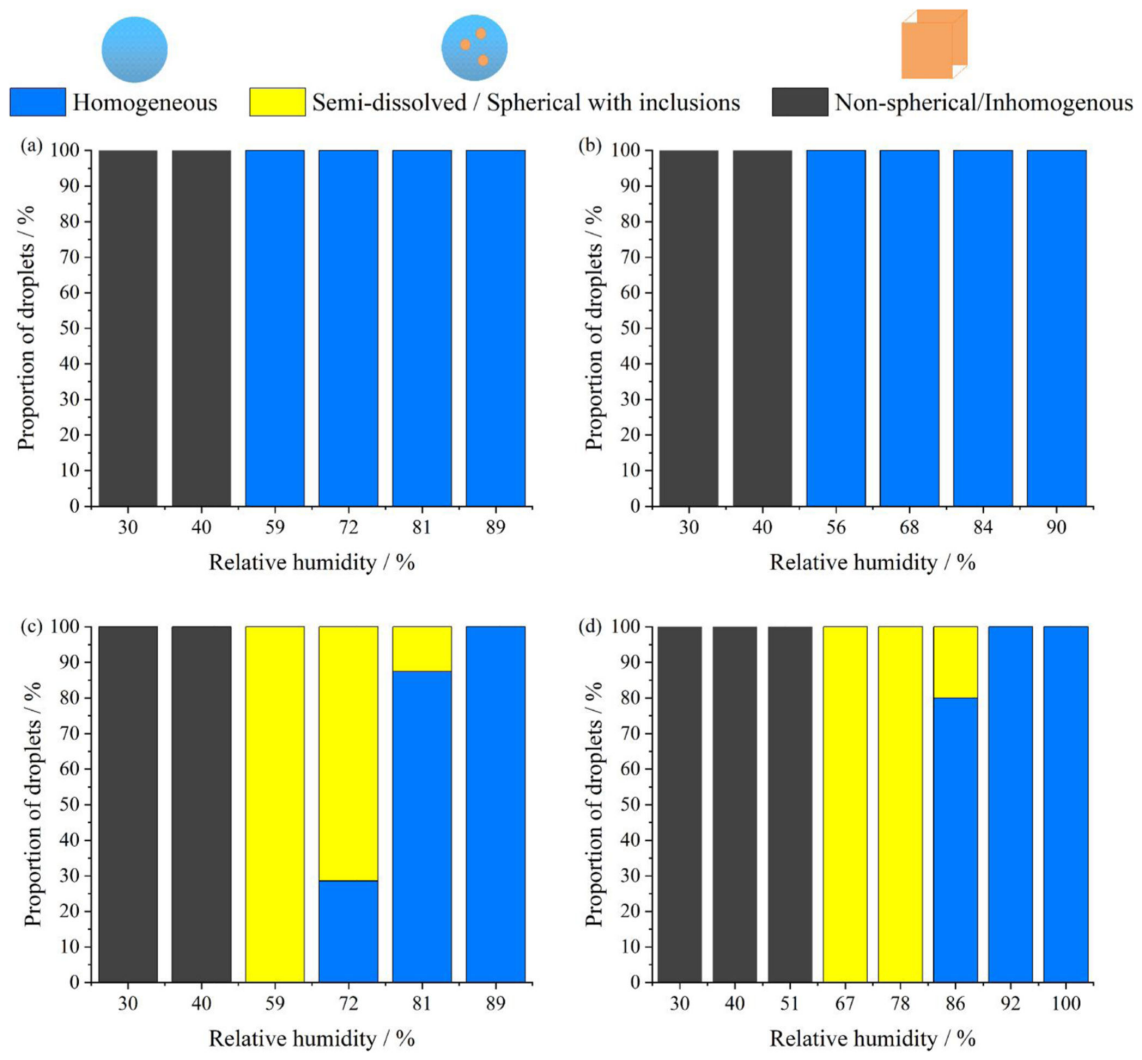
RH-dependent phase behavior of levitated aerosol of (a) NaCl (of 0.1 MFS), (b) AS (exclude mucin, DMEM), (c) DMEM (with 10% FBS), and (d) MEM (with 2% FBS) solution from previous study (Oswin et al. 2022). Blue, dark grey, and yellow indicate homogeneous, non-spherical-inhomogeneous, semi-dissolved (spherical with inclusions) phase respectively.

Nucleation is a kinetically controlled process that is more facile at higher solute supersaturation. The supersaturation can be estimated from the variation in the equilibrium composition with water activity. Different efflorescence RHs are observed for different solution droplets (Figure 7). MEM effloresced at ~55% RH (Oswin et al. 2022), while NaCl, AS and DMEM effloresced at 40 − 45%. The measurements we report are designed to capture the dynamic changes in phase and timescales of droplet transformation during evaporation and drying, simulating the processes occurring following exhalation. This is an important distinction from previous work that has examined the equilibrium response in steady-state particle moisture content and phase achieved following step changes in RH (see, for example, Davies et al. 2021). Directly probing the processes through to equilibration, rather than just the equilibrium state, allows investigations of the influence of dynamic properties, such as inhomogeneities in composition and evaporative cooling, on phase behavior. This approach is central to our approach where we inject a droplet into a low RH environment and monitor the dynamic change rather than the steady state.

During the evaporation of NaCl and AS (mucin-free) droplets studied here, only two phases are observed, droplets that remain homogenous when the ambient RH is above the efflorescence RH and effloresce when the ambient RH is below the efflorescence RH (Figure 7a and b). However, the two cell culture media DMEM and MEM (with FBS) both exhibit three distinct phases. DMEM and MEM droplets remain homogenous when RH is above ~85% and form a non-spherical inhomogeneous particle when RH is below 45%. A semi-dissolved (spherical with inclusions) phase is observed between 45% to 85% RH, which is within the typical environmental humidity range. This observation is in agreement with the study from Huynh et al. (2022), where three distinct phases are observed in the DMEM droplet over a range of RH; they observed the semisolid phase state at ~53% RH using coalescence and microscopy imaging characterization methods.

### 3.3 The effect of mucin on droplet evaporation and phase behavior at low RH

**Figure 8:**
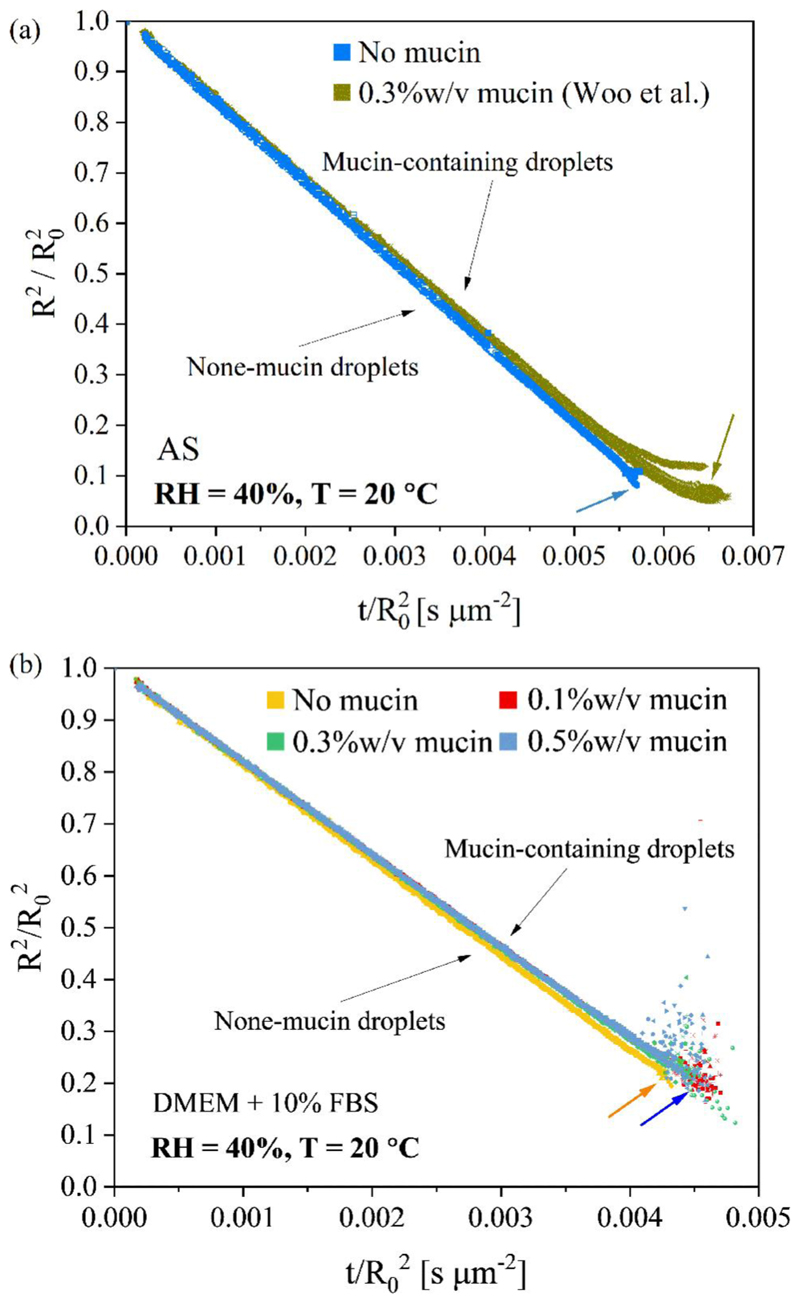
Comparison of mucin-containing and non-mucin droplets for (a) AS and (b) DMEM.

Mucin glycoproteins (mucins) are the major macro-molecular constituents of epithelial mucus and have long been investigated in health and disease research (Wardzala et al. 2022; Rose and Voynow 2006). In this study, porcine type II mucin serve as a surrogate for the secretory gel-forming mucins in saliva upregulated during respiratory diseases. Clinically relevant concentrations of mucin were added to DMEM and the impact of the corresponding change in phase behavior on the airborne survival of mouse hepatitis virus (MHV) was investigated; a transient delay in the loss of viral infectivity at 40% RH was observed (Alexander et al. 2022). In this study, we have added mucin to both DMEM and AS solution, and 20 droplets of each solution were levitated and dried at 40% RH, 20 °C in the CK-EDB (Figure 8a and b). The EDB measurements show that adding mucin to both AS and DMEM droplets only marginally limits the rate of water evaporation in the latter stages of drying when compared with non-mucin droplets. However, the evaporation rates of droplets containing different mucin concentrations are comparable, indicating that the impact of mucin is not strongly dependent on concentration. A marginal delay in the crystallization times is also observed for the mucin-containing droplets when compared to droplets devoid of mucin. This may be due to the formation of a viscous mucin surface film, which affects the mass transport and crystallization dynamics.

**Figure 9:**
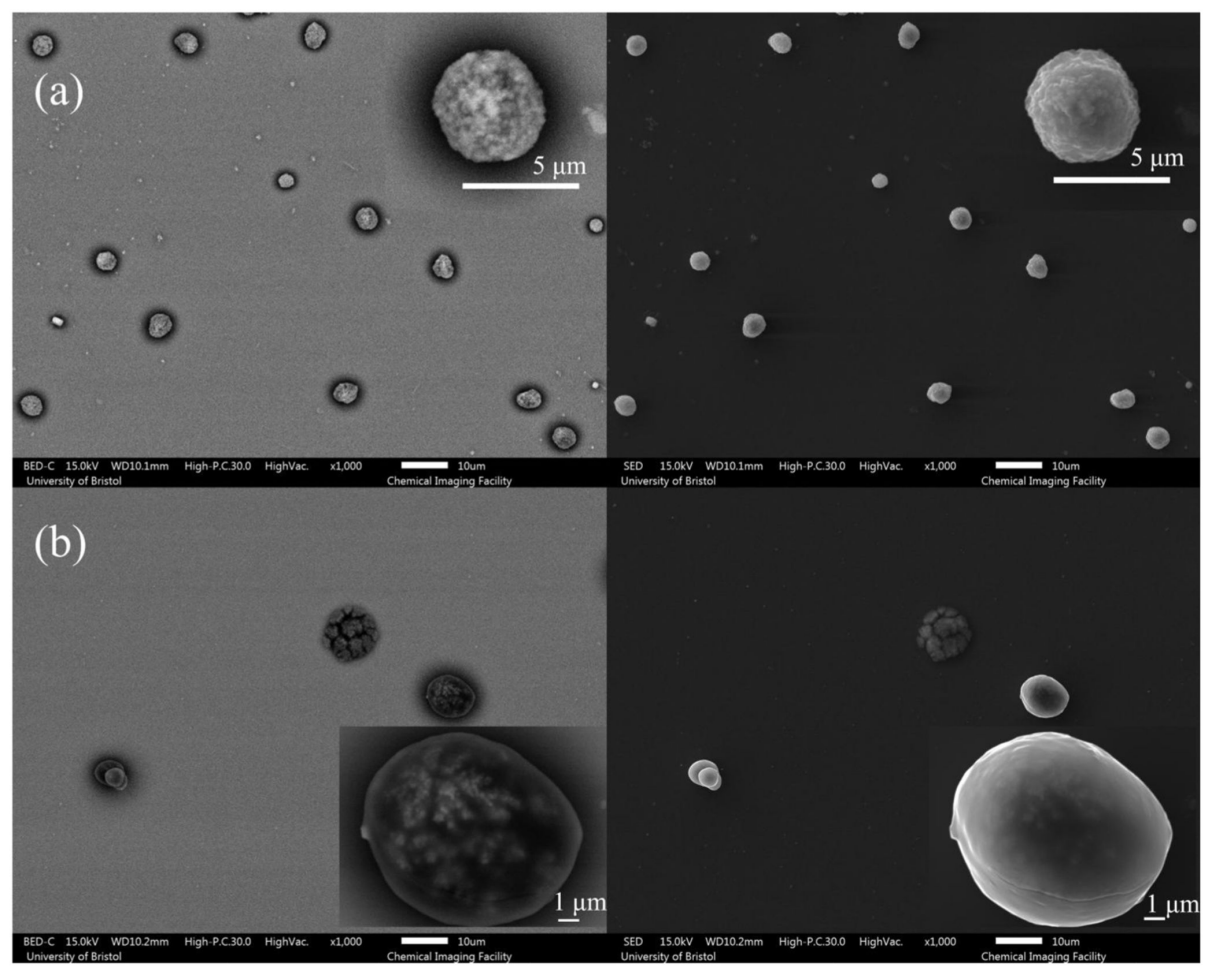
SEM images of mucin-containing (a) AS and (b) DMEM droplets drying at 40% RH. Note, that each figure is composed of the images from the backscattered electron detector (BED) (left) as well as the secondary electron detector (SED) (right).

The influence of mucin on the morphology of dried particles can be observed from the SEM images (Figure 9a and b). In both cases, AS and DMEM solution droplets with mucin tend to form more spherical particles and surface enrichment is apparent; droplets devoid of mucin result in dried particles with more irregular shapes. AS droplets without mucin tend to present non-spherical and hollow structured particles (Figure 2b). DMEM dried particles (Figure 2c) have a smooth surface, which is likely due to the existence of organics (e.g., glucose) compared to AS particles (Figure 9a and b). Crystals are formed within DMEM particles, and the crystals are likely surrounded by organics; for AS particles, the mucin (organic part) is located in the gaps between crystals (Figure 9a). The DMEM dried particles without mucin appear to have a larger number of crystals formed on the surface while the ones containing mucin showed less surface crystallization (Figure 2b and c). It is also expected that the organic components of the solution will increase the viscosity of the solution which will limit solute diffusion and consequently impact upon the crystal growth and particle morphology.

**Figure 10:**
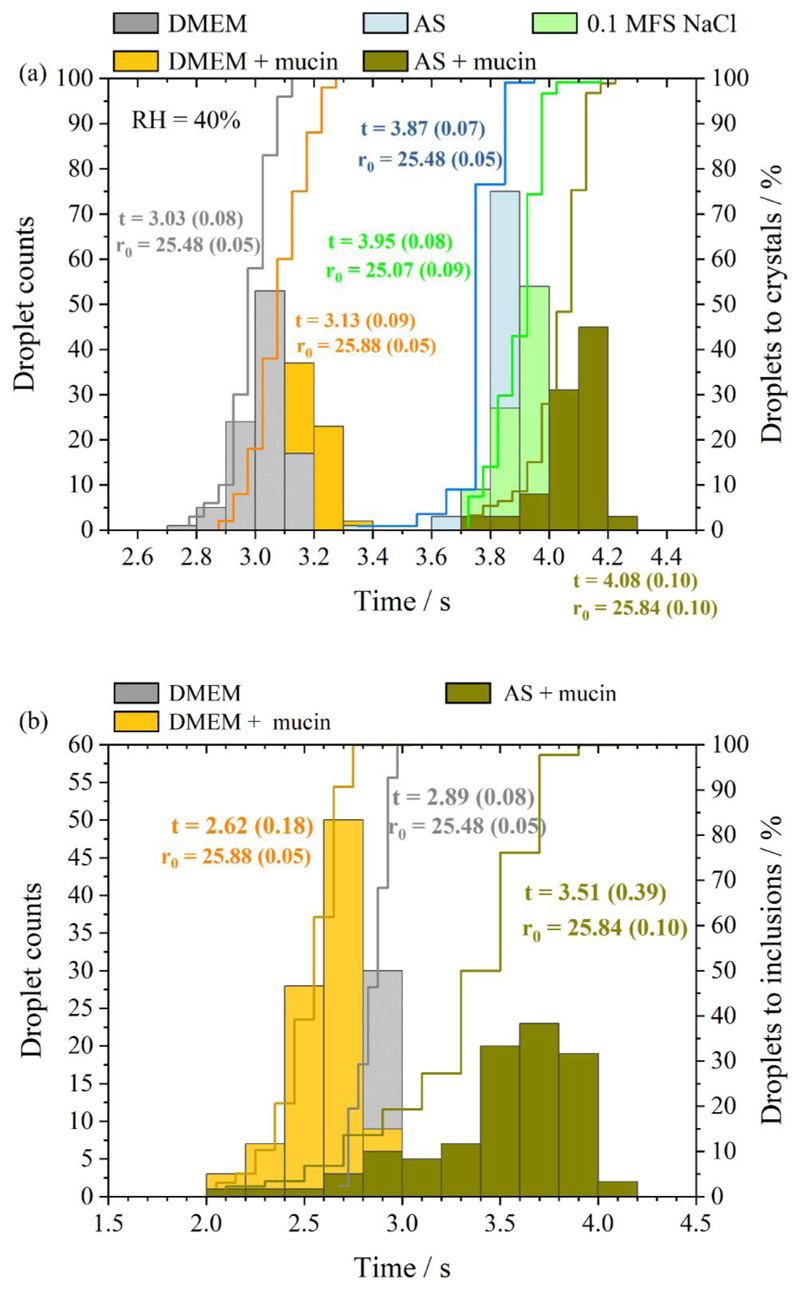
Time-dependent phase behavior across ~100 particles of NaCl, AS and DMEM solution droplets drying at 40% RH showing (a) time to reach crystallization; (b) time to inclusion body formation. Mucin concentration in each mucin-containing solutions: DMEM contains 5 g/L (or 0.5% *w/v*), AS contains 3 g/L (or 0.3% *w/v*) following (Woo et al. 2010).

The time taken for an evaporating aqueous droplet of NaCl, AS or DMEM solution to effloresce and form inclusion bodies was recorded for over 100 droplets at the same gas-phase temperature (20 °C) and relative humidity (40% RH). The impact of mucin content on the transition in particle phase during evaporation (Figure 10a and b) was investigated. Interestingly, the crystallization time for NaCl droplets (3.95 s) is comparable to AS droplets, while DMEM crystalized about 1 s earlier than AS and NaCl particles. Adding mucin marginally delayed the crystallization time for both AS and DMEM solution droplets, with a 0.21 s delay for AS droplets and a 0.1 s delay for DMEM droplets (Figure 10a). Mucin promoted heterogenous phase change during aerosol evaporation in both AS and DMEM droplets (Figure 10b). Three paired *t*-tests were conducted for both AS/AS with mucin and DMEM/DMEM with mucin to examine the statistical significance between the pairs for both times to reach crystallization and inclusions (Table S7). The statistical results shows that the delay in time to reach crystallization for droplets with mucin for AS and DMEM solution is statistically significant compared to the mucin-free droplets (*p* < 0.001). In addition, adding mucin significantly decreased the time to reach the inclusion phase (*p* < 0.001).

The mucin concentration profile through the droplet may be variable between droplets, but it is possible to observe the overall trends of the phase change by measuring 100 droplets, benefiting from the highly reproducible drying measurement carried out in the CK-EDB. Hence, we propose that mucin influences the times at which the phase change of the aerosols occurs. Even though this delay is marginal, it leads to greater loss of water before phase change occurs, particles that may be structured in different ways, and a level of residual water that may be kinetically limited in release. All of these factors can promote sustained infectivity. The presence of mucin in aerosol droplets sustains coronavirus infectivity in the short term (with 2 min of droplet production) but has little effect in the longer term (≥5 min) (Alexander et al. 2022). Indeed, viral survival in droplets containing higher mucin concentrations show higher mean infectivity for short timescale levitations (30 s) at 40% RH in DMEM droplets. This difference has been ascribed to changes in the phase behavior between mucin-containing and mucin-free droplets. This again underlines the change in phase behavior is crucial to understanding the changes in infectivity compared to the droplet evaporation rate and delayed particle crystallization time due to slower water diffusivity.

## 4 Conclusions

We have investigated the microphysical characteristics of three surrogate solution droplets for simulating exhaled aerosols and also for laboratory studies of the airborne survival of SARS-CoV-2 and the Mouse Hepatitis Virus. The evaporation kinetics and equilibrium thermodynamics of NaCl, AS, and DMEM solution droplets were measured and compared with MEM solution droplet by the CK-EDB technique, revealing trends in evaporation behavior and resultant phase change depending upon the drying conditions and initial conditions. The phase transformation and the effect of mucin concentration upon evaporation are also reported. Overall, water transport is crucial to understanding the phase behavior and corresponding viral and bacterial survival in respiratory aerosol. Phase separation of salts and organic material was observed in dried particles, and the complex phase behavior in the environmentally relevant humidity range is likely the reason for the increased variability in microbial viability in the aerosol phases (Alexander et al. 2022; Oswin et al. 2021, 2022). Mucin delayed the water transport and promoted phase change to occur at higher water activities during aerosol evaporation, with timescales consistent with the timescale of the effect of mucin on viral infectivity loss.

The microphysical measurements from this study can contribute to the estimation of phase behavior, size distribution, and deposition efficiency of different respiratory secretions upon inhalation and thus help estimate infection sites and dose. The compositional change holds the potential to reflect the true nature of human secretions such as oral and nasal and using surrogates of human secretions can shed light on the mechanism of viral survival in the aerosol phase. Results from this work can inform the laboratory choices of bioaerosol survival matrix and also inform the implementation of strategies to mitigate the spread of diseases such as COVID-19. There remains a need to understand CO_2_ partitioning and its corresponding effects on pH change in respiratory droplets (Haddrell et al. 2023; Oswin et al. 2022), and also the need to explore the corresponding reduction in pH from condensable acidic species (Klein et al. 2022). The microphysics measurement of this bicarbonate/CO_2_ partitioning effect and the impact of droplet pH will be addressed in a future publication.

## Supplementary Material

Table S1-S6, Fig. S1
