# Supplementary material for: The microphysics of surrogates of exhaled aerosols from the upper respiratory tract
Source: Aerosol Sci Technol. Author manuscript; Available in PMC 2025 Apr 15. (PMC7617586; doi:10.1080/02786826.2023.2299214)
Supplement: Table S1-S6, Fig. S1 [file EMS204100-supplement-Table_S1_S6__Fig__S1.docx]

**Supplemental Information**

**The Microphysics of Surrogates of Exhaled Aerosols from the Upper Respiratory Tract**

Jianghan Tian^1^, Robert W. Alexander^2^, Daniel A. Hardy^1^, Thomas G. Hilditch^1^, Henry P. Oswin^3^, Allen E. Haddrell^1^, Jonathan P. Reid^1, *^

^1^School of Chemistry, University of Bristol, Bristol, BS8 1TS, United Kingdom

^2^School of Cellular and Molecular Medicine, University of Bristol, Bristol BS8 1TD, United Kingdom

^3^School of Earth and Atmospheric Sciences, Queensland University of Technology, Brisbane, QLD 4001, Australia

^*^Corresponding authors: j.p.reid@bristol.ac.uk

**Table S1:** Compositions of artificial saliva, adapted from (Woo et al. 2010).

| **Components** | **Concentration (g/L)** |
| --- | --- |
| MgCl_2_∙6H_2_O | 0.037 |
| CaCl_2_∙2H_2_O | 0.15 |
| NaHCO_3_ | 0.42 |
| KH2PO_4_ | 0.21 |
| K2HPO_4_ | 0.43 |
| NH_4_Cl | 0.11 |
| KSCN | 0.19 |
| (NH_2_)_2_CO | 0.12 |
| NaCl | 0.88 |
| KCl | 1.04 |
| (Mucin) | 3 |
| (DMEM) | 1 mL |
| Deionised water | 979 mL |

**Table S2:** Compositions of DMEM.

| **Components** | **Concentration (g/L)** |
| --- | --- |
| **Inorganic salts** | |
| Calcium chloride (CaCl_2_) (anhyd.) | 0.2 |
| Ferric nitrate (Fe(NO_3_)_3_∙9H2O) | 0.0001 |
| Magnesium sulfate (MgSO_4_) (anhyd.) | 0.09767 |
| Potassium chloride (KCl) | 0.4 |
| Sodium bicarbonate (NaHCO_3_) | 3.7 |
| Sodium chloride (NaCl) | 6.4 |
| Sodium phosphate monobasic (NaH_2_PO_4_-H_2_O) | 0.125 |
| **Amino acids** | |
| Glycine | 0.03 |
| L-Arginine hydrochloride | 0.084 |
| L-Cystine 2HCl | 0.063 |
| L-Glutamine | 0.584 |
| L-Histidine hydrochloride-H_2_O | 0.042 |
| L-Isoleucine | 0.105 |
| L-Leucine | 0.105 |
| L-Lysine hydrochloride | 0.146 |
| L-Methionine | 0.03 |
| L-Phenylalanine | 0.066 |
| L-Serine | 0.042 |
| L-Threonine | 0.095 |
| L-Tryptophan | 0.016 |
| L-Tyrosine disodium salt dihydrate | 0.104 |
| L-Valine | 0.094 |
| **Vitamins** | |
| Choline chloride | 0.004 |
| D-Calcium pantothenate | 0.004 |
| Folic Acid | 0.004 |
| Niacinamide | 0.004 |
| Pyridoxine hydrochloride | 0.004 |
| Riboflavin | 0.0004 |
| Thiamine hydrochloride | 0.004 |
| i-Inositol | 0.0072 |
| **Other components** | |
| D-Glucose (Dextrose) | 4.5 |
| Phenol red | 0.015 |

**Table S3:** Compositions of MEM.

| **Components** | **Concentration (g/L)** |
| --- | --- |
| **Amino acids** | |
| L-Alanyl-Glutamine | 0.406 |
| L-Arginine hydrochloride | 0.126 |
| L-Cystine | 0.024 |
| L-Histidine hydrochloride-H_2_O | 0.042 |
| L-Isoleucine | 0.052 |
| L-Leucine | 0.052 |
| L-Lysine hydrochloride | 0.073 |
| L-Methionine | 0.015 |
| L-Phenylalanine | 0.032 |
| L-Threonine | 0.048 |
| L-Tryptophan | 0.01 |
| L-Tyrosine | 0.036 |
| L-Valine | 0.046 |
| **Vitamins** | |
| Choline chloride | 0.001 |
| D-Calcium pantothenate | 0.001 |
| Folic Acid | 0.001 |
| Niacinamide | 0.001 |
| Pyridoxal hydrochloride | 0.001 |
| Riboflavin | 0.0001 |
| Thiamine hydrochloride | 0.001 |
| i-Inositol | 0.002 |
| **Inorganic salts** | |
| Calcium Chloride (CaCl_2_-2H_2_O) | 0.264 |
| Magnesium Sulfate (MgSO_4_-7H_2_O) | 0.2 |
| Potassium Chloride (KCl) | 0.4 |
| Sodium Bicarbonate (NaHCO_3_) | 2.2 |
| Sodium Chloride (NaCl) | 6.8 |
| Sodium Phosphate monobasic (NaH_2_PO_4_-2H_2_O) | 0.158 |
| **Other components** | |
| D-Glucose (Dextrose) | 1 |
| Phenol Red | 0.01 |

**Table S4**: The descriptive summary of the data and ANOVA test in Figure 1.

| **(a) NaCl (0.1 MFS)** | | | | | |
| --- | --- | --- | --- | --- | --- |
| RH (%) | Initial radius (average)  (μm) | Equilibrium (or crystal) radius (average) (μm) | Initial radius (standard deviation) | Equilibrium (or crystal) radius (standard deviation) | Measured droplet number |
| 90 | 25.31 | 21.88 | 0.02 | 0.28 | 10 |
| 81 | 25.15 | 18.37 | 0.11 | 0.12 | 10 |
| 68 | 25.51 | 16.44 | 0.01 | 0.02 | 10 |
| 52 | 25.28 | 14.43 | 0.07 | 0.03 | 10 |
| 40 | 25.34 | 14.05 | 0.07 | 0.04 | 10 |
| **(b) AS** | | | | | |
| RH (%) | Initial radius (average) (μm) | Equilibrium (or crystal) radius (average) (μm) | Initial radius (standard deviation) | Equilibrium (or crystal) radius (standard deviation) | Measured droplet number |
| 90 | 25.89 | 6.42 | 0.05 | 0.06 | 10 |
| 84 | 23.79 | 5.38 | 0.01 | 0.08 | 10 |
| 68 | 25.97 | 5.12 | 0.14 | 0.15 | 10 |
| 40 | 25.56 | 8.20 | 0.09 | 0.61 | 10 |
| **(c) DMEM** | | | | | |
| RH  (%) | Initial radius (average) (μm) | Equilibrium (or crystal) radius (average) (μm) | Initial radius (standard deviation) | Equilibrium (or crystal) radius (standard deviation) | Measured droplet number |
| 89 | 25.39 | 10.68 | 0.19 | 0.76 | 10 |
| 81 | 25.85 | 8.84 | 0.73 | 0.26 | 10 |
| 72 | 25.64 | 8.76 | 0.21 | 0.65 | 10 |
| 59 | 25.68 | 8.31 | 0.17 | 0.32 | 8 |
| 40 | 25.56 | 11.77 | 0.03 | 0.61 | 10 |

For the 90% RH final equilibrium size ANOVA test:

| Surrogates | Compared Groups | Mean Difference | Std. Error | Sig. | 95% Confidence Interval | |
| --- | --- | --- | --- | --- | --- | --- |
|  |  |  |  |  | Lower Bound | Upper Bound |
| NaCl | 2 | 15.45700* | 0.21061 | <.001 | 14.9348 | 15.9792 |
|  | 3 | 11.19500* | 0.21061 | <.001 | 10.6728 | 11.7172 |
| AS | 1 | -15.45700* | 0.21061 | <.001 | -15.9792 | -14.9348 |
|  | 3 | -4.26200* | 0.21061 | <.001 | -4.7842 | -3.7398 |
| DMEM | 1 | -11.19500* | 0.21061 | <.001 | -11.7172 | -10.6728 |
|  | 2 | 4.26200* | 0.21061 | <.001 | 3.7398 | 4.7842 |

* The mean difference is significant at the 0.05 level.

Number 1, 2, 3 in the “Compared Groups” indicates NaCl, AS, and DMEM respectively.

For the 40% RH final (crystallise) size ANOVA test:

| Surrogates | Compared Groups | Mean Difference | Std. Error | Sig. | 95% Confidence Interval | |
| --- | --- | --- | --- | --- | --- | --- |
|  |  |  |  |  | Lower Bound | Upper  Bound |
| NaCl | 2 | 5.85400* | 0.22389 | <.001 | 5.2989 | 6.4091 |
|  | 3 | 2.28500* | 0.22389 | <.001 | 1.7299 | 2.8401 |
| AS | 1 | -5.85400* | 0.22389 | <.001 | -6.4091 | -5.2989 |
|  | 3 | -3.56900* | 0.22389 | <.001 | -4.1241 | -3.0139 |
| DMEM | 1 | -2.28500* | 0.22389 | <.001 | -2.8401 | -1.7299 |
|  | 2 | 3.56900* | 0.22389 | <.001 | 3.0139 | 4.1241 |

* The mean difference is significant at the 0.05 level.

Number 1, 2, 3 in the “Compared Groups” indicates NaCl, AS, and DMEM respectively.

**Table S5:** The parameter used for fitting hygroscopic curves of AS, ASS and DMEM in Figure 2a. The fitted model is based on the Equation 17 in (Kreidenweis et al. 2005).

$$GF={[1+(a+b\cdot a_{w}+c\cdot a_{w}^{2})\cdot\frac{a_{w}}{1-a_{w}}]}^{\frac{1}{3}}$$

The a, b, c for each medium is:

| **Coefficients** | ***a*** | ***b*** | ***c*** |
| --- | --- | --- | --- |
| **Artificial saliva (AS)** | 1.7331 | -3.0512 | 1.5986 |
| **Artificial stimulated saliva (ASS)** | 1.63 | -2.6833 | 1.7887 |
| **Dulbecco’s modified Eagle’s medium (DMEM)** | 3.5983 | -4.323 | 1.3895 |

**Table S6:** The modelled relationship between aw, MFS and density for saliva (from Walker et al. (2021)) and DMEM droplets derived from EDB measurements.

$$MFS=a+\left( b{\times a_{w}}^{1} \right)+\left( c{\times a_{w}}^{2} \right)+\left( d{\times a_{w}}^{3} \right)+\left( e{\times a_{w}}^{4} \right)$$

$$Density= f+\left( g{\times MFS}^{0.5} \right)+\left( h{\times MFS}^{1} \right)+\left( i{\times MFS}^{1.5} \right)+(j\times{MFS}^{2})$$

|  | ***a*** | ***b*** | ***c*** | ***d*** | ***e*** |
| --- | --- | --- | --- | --- | --- |
| **AS** | 1 | 0.13414 | -4.73918 | 10.23153 | -6.62626 |
| **DMEM** | 1 | -1.4314 | 2.0361 | -1.5724 | 0 |
|  | ***f*** | ***g*** | ***h*** | ***i*** | ***j*** |
| **AS** | 995.78 | 262.92 | -606.15 | 1135.53 | 0 |
| **DMEM** | 998.21 | -22.25 | 840.08 | -1117.7 | 1151.6 |

**Table S7:** The paired t-test for Figure 10.

I. Adding mucin increased the crystallisation time.

- For DMEM:

|  | *DMEM* | *DMEM+mucin* |
| --- | --- | --- |
| Mean | 3.03203 | 3.12886 |
| Variance | 0.005702938 | 0.007831778 |
| Observations | 100 | 100 |
| Pooled Variance | 0.006767358 |  |
| Hypothesized Mean Difference | 0 |  |
| df | 198 |  |
| t Stat | -8.323104501 |  |
| P(T<=t) one-tail | 6.92387E-15 |  |
| t Critical one-tail | 1.652585784 |  |
| P(T<=t) two-tail | 1.38477E-14 | *p* < 0.001 |
| t Critical two-tail | 1.972017478 |  |

- For AS:

|  | *AS* | *AS+mucin* |
| --- | --- | --- |
| Mean | 3.870576577 | 4.079645161 |
| Variance | 0.004881737 | 0.009907558 |
| Observations | 111 | 93 |
| Pooled Variance | 0.007170725 |  |
| Hypothesized Mean Difference | 0 |  |
| df | 202 |  |
| t Stat | -17.56285576 |  |
| P(T<=t) one-tail | 7.80865E-43 |  |
| t Critical one-tail | 1.652431964 |  |
| P(T<=t) two-tail | 1.56173E-42 | *p* < 0.001 |
| t Critical two-tail | 1.971777385 |  |

II. Adding mucin decreased the time to reach inclusion phase.

|  | *DMEM* | *DMEM + mucin* |
| --- | --- | --- |
| Mean | 2.89397561 | 2.619680412 |
| Variance | 0.006716874 | 0.031051761 |
| Observations | 41 | 97 |
| Pooled Variance | 0.023894442 |  |
| Hypothesized Mean Difference | 0 |  |
| df | 136 |  |
| t Stat | 9.525943742 |  |
| P(T<=t) one-tail | 4.28418E-17 |  |
| t Critical one-tail | 1.656134988 |  |
| P(T<=t) two-tail | 8.56836E-17 | *p* < 0.001 |
| t Critical two-tail | 1.977560777 |  |


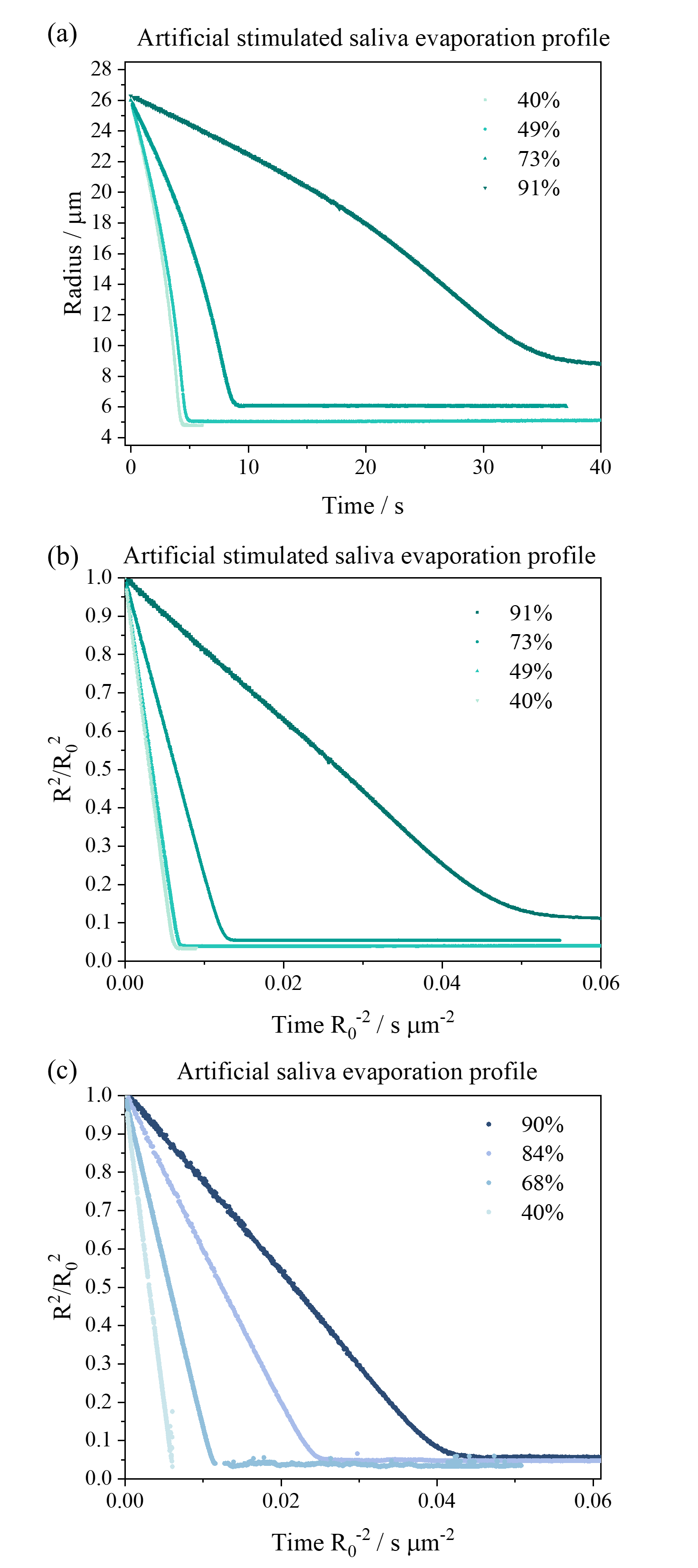


**Figure S1:** The hygroscopicity profile of surrogate systems and the comparison of evaporation profile of artificial saliva and artificial stimulated saliva.

**References**

Kreidenweis, S. M., K. Koehler, P. J. DeMott, A. J. Prenni, C. Carrico, and B. Ervens. 2005. “Water Activity and Activation Diameters from Hygroscopicity Data - Part I: Theory and Application to Inorganic Salts.” *Atmospheric Chemistry and Physics* 5 (5): 1357–70. https://doi.org/10.5194/acp-5-1357-2005.

Walker, Jim S., Justice Archer, Florence K. A. Gregson, Sarah E. S. Michel, Bryan R. Bzdek, and Jonathan P. Reid. 2021. “Accurate Representations of the Microphysical Processes Occurring during the Transport of Exhaled Aerosols and Droplets.” *ACS Central Science* 7: 200–209. https://doi.org/10.1021/acscentsci.0c01522.

Woo, Myung Heui, Yu Mei Hsu, Chang Yu Wu, Brian Heimbuch, and Joseph Wander. 2010. “Method for Contamination of Filtering Facepiece Respirators by Deposition of MS2 Viral Aerosols.” *Journal of Aerosol Science* 41 (10): 944–52. https://doi.org/10.1016/j.jaerosci.2010.07.003.
